# Soil Texture, Sampling Depth and Root Hairs Shape the Structure of ACC Deaminase Bacterial Community Composition in Maize Rhizosphere

**DOI:** 10.3389/fmicb.2021.616828

**Published:** 2021-02-04

**Authors:** Lucie Gebauer, Marie-Lara Bouffaud, Minh Ganther, Bunlong Yim, Doris Vetterlein, Kornelia Smalla, François Buscot, Anna Heintz-Buschart, Mika T. Tarkka

**Affiliations:** ^1^Helmholtz Centre for Environmental Research, Halle, Germany; ^2^Julius Kühn-Institute, Braunschweig, Germany; ^3^Institute of Agricultural and Nutritional Sciences, Martin Luther University Halle-Wittenberg, Halle, Germany; ^4^German Centre for Integrative Biodiversity Research (iDiv) Halle-Jena-Leipzig, Leipzig, Germany

**Keywords:** ethylene, rhizosphere, soil, root, plant-microbe interactions, PGPR, crop

## Abstract

Preservation of the phytostimulatory functions of plant growth-promoting bacteria relies on the adaptation of their community to the rhizosphere environment. Here, an amplicon sequencing approach was implemented to specifically target microorganisms with 1-aminocyclopropane-1-carboxylate deaminase activity, carrying the *acdS* gene. We stated the hypothesis that the relative phylogenetic distribution of *acdS* carrying microorganisms is affected by the presence or absence of root hairs, soil type, and depth. To this end, a standardized soil column experiment was conducted with maize wild type and root hair defective *rth3* mutant in the substrates loam and sand, and harvest was implemented from three depths. Most *acdS* sequences (99%) were affiliated to Actinobacteria and Proteobacteria, and the strongest influence on the relative abundances of sequences were exerted by the substrate. *Variovorax, Acidovorax*, and *Ralstonia* sequences dominated in loam, whereas *Streptomyces* and *Agromyces* were more abundant in sand. Soil depth caused strong variations in *acdS* sequence distribution, with differential levels in the relative abundances of *acdS* sequences affiliated to *Tetrasphaera, Amycolatopsis*, and *Streptomyces* in loam, but *Burkholderia*, *Paraburkholderia*, and *Variovorax* in sand. Maize genotype influenced the distribution of *acdS* sequences mainly in loam and only in the uppermost depth. Variovorax *acdS* sequences were more abundant in WT, but *Streptomyces, Microbacterium*, and *Modestobacter* in *rth3* rhizosphere. Substrate and soil depth were strong and plant genotype a further significant single and interacting drivers of *acdS* carrying microbial community composition in the rhizosphere of maize. This suggests that maize rhizosphere *acdS* carrying bacterial community establishes according to the environmental constraints, and that root hairs possess a minor but significant impact on *acdS* carrying bacterial populations.

## Introduction

Maize (*Zea mays* L.) is one of the most important staple crops worldwide in terms of agronomy and global economic impact (Food and Agriculture Organization of the United Nations, 2019). Various maize cultivars are grown in different climatic zones, and maize is used as fodder, for human consumption, or for industrial products such as biofuel. Maize cultivation requires intensive management and high levels of resources, and there is a growing need to find sustainable practices (Akinnifesi et al., 2010; Shiferaw et al., 2011; Sauerbrei et al., 2014).

Research on the development of maize root system and rhizosphere microbiome is steadily increasing, since improving root growth and the efficiency of plant beneficial microbes can support maize growth under normal and stress conditions (Lynch, 2015; Brisson et al., 2019). In order to support phytostimulatory plant-microbe interactions for sustainable maize production, the first step is to characterize maize rhizosphere communities. Investigations on the bacterial 16S rRNA microbiome indicate that like in other plants, maize rhizosphere bacterial community composition differs from that in bulk soil (Bouffaud et al., 2014). They also indicate that field conditions and crop rotation (Benitez et al., 2017), pathogens (Saravanakumar et al., 2017), soil properties and plant age (Bouffaud et al., 2012; Peiffer et al., 2013) interact to drive the maize rhizosphere microbiome composition. Further modifications are caused by maize genotypes (Bouffaud et al., 2014; Walters et al., 2018) and rhizodeposition (Cotton et al., 2019).

Plant growth promoting rhizobacteria (PGPR) are a ubiquitous and species-rich group of bacteria (Antoun and Prévost, 2005; Renoud et al., 2020) that have a considerable potential to support agricultural practice by their positive influence on plant nutrition, growth, abiotic stress and disease resistance (Egamberdiyeva, 2007; Tak et al., 2013; Timmusk et al., 2017). Enriching PGPR populations represents an attractive means for future agricultural technologies to maintain yields while reducing mineral fertilization (Gamalero and Glick, 2015; Brisson et al., 2019). Rhizosphere PGPR community is a subset of the bulk soil community (Li et al., 2012), and its structure, characterized by barcoding their 16S rRNA, is also modulated by the drivers described earlier for whole bacterial microbiome (Roesch et al., 2008; Coelho et al., 2009; Bouffaud et al., 2018; Long et al., 2018; Renoud et al., 2020). Variable efficacy of PGPR populations under field conditions, where several environmental factors act simultaneously and host plant species vary, indicates that limited knowledge on their community response to the environment and the organismal interactions in the root-soil interface are perhaps the main factors that hampers the widespread use of the PGPR (Timmusk et al., 2017).

Many PGPR have the potential to support growth of plants by altering their hormonal balance. This takes place either by affecting plant hormone production, exuding plant hormones or producing hormone precursor degrading enzymes (Shahzad et al., 2013; Rozier et al., 2017). Plant hormone related PGPR activities can support root growth, since root system architecture is adapted through the modulation of phytohormone levels (Vacheron et al., 2013) in response to environmental cues. For example, auxins such as indole-3-acetic acid modulate root elongation and induce lateral root formation, and indeed, the PGPR may alter root systems by either producing auxins, or inducing *in planta* auxin production (Sukumar et al., 2013). The effect of the plant hormone ethylene on root growth is largely mediated through interactions with auxin (Ivanchenko et al., 2008), via the regulation of auxin biosynthesis or local auxin distribution (Růžička et al., 2007), and increased ethylene levels essentially lead to the inhibition of root elongation and increased production of root hairs (Tanimoto et al., 1995). In this context, some bacteria deaminate the precursor of ethylene, 1-aminocyclopropane-1-carboxylate (ACC), and the ACC deaminase activity lowers ethylene levels and alleviates the repressive effect of ethylene on root elongation that leads to improved nutrient acquisition and water uptake capacity. Due to these effects on the host plant, the ACC deaminase positive bacteria support plants grown under fair as well as stressful conditions (Glick, 2005; Jaemsaeng et al., 2018; Yoolong et al., 2019).

Our earlier data from a maize field revealed, that the bacteria carrying the *acdS* gene (*acdS+* bacteria) are more abundant in the rhizosphere than in the bulk soil, and amplicon sequencing analysis of the *acdS+* community composition revealed that it is affected by maize genotype (Bouffaud et al., 2018). It was also identified, that maize growth stage further affects *acdS+* community composition, and it represents an interacting driver to the field site. During the flowering stage, the *acdS+* diversity level and community composition varied across three field sites with luvisol, fluvic cambisol, and calcisol soil types, but not during the early, six leaf stage of maize development (Renoud et al., 2020). It is not yet established whether depth within the soil or the production of root hairs affect the structure of *acdS+* communities. Soil depth in the field affects the distribution of available nutrients, water and oxygen, and subsequently, different soil depths in the field show distinct assemblies of bacteria (Fierer et al., 2003). Root hairs are protrusions of epidermal cells that extend to the rhizosphere and change rhizosphere properties by taking up nutrients and by exudation of organic compounds and enzymes (Marschner et al., 2011). By using barley root hair formation and elongation mutants and applying 16S rRNA gene amplicon sequencing, Robertson-Albertyn et al. (2017) associated the presence of the root hairs to the depletion of the relative abundances of some and the increase of other bacterial taxa, and observed that the root hair mutants triggered a pronounced effect on the bacterial community in a substrate with lower organic matter content.

In the frame of the German Science Foundation’s priority program “Rhizosphere Spatiotemporal Organization” (Vetterlein et al., 2020a) we implemented a soil column experiment (Vetterlein et al., 2020b) in loam and sand substrates to assess the effects of substrate, the depths where the samples were taken, i.e., depth, and root hair presence/absence on *acdS+* community composition. Three hypotheses were stated: (i) the relative phylogenetic distribution of the *acdS+* community structure is affected by substrate, root hair formation, and depth, (ii) *acdS+* genera that are differentially abundant between loam and sand will include genera whose relative abundance have been shown to be affected by field conditions, and, (iii) the influence of root hair presence/absence is different in loam than sand.

## Materials and Methods

### Soil Preparation and Plant Material

The design of the soil column experiments was described in detail by Vetterlein et al. (2020b). Briefly, loamy soil (L) substrate, which was classified as Haplic Phaeozem, consisted of 32.5% sand, 47.9% silt, and 19.5% clay (Supplementary Table 1). Inorganic carbon was not detectable; therefore, total carbon was assumed to represent the organic carbon content with 8.6 g kg^–1^ C_org_. The nitrogen content was 0.84 g kg^–1^ N_org_, resulting in a C:N ratio of 10.2. Soil pH (CaCl_2_) was 6.4. The substrate *sand* (S) was obtained by mixing L with quartz sand in 83.3%:16.7% ratio. The reason for mixing loamy soil (L) substrate with quartz sand was to yield two substrates with different textures but the same microbial inoculate (of the original soil). To enable similar nutrient uptake of maize on both substrates, loam and sand were fertilized differently according to Vetterlein et al. (2020b). Like for barley, root hair formation, and elongation mutants have also been constructed for maize (Hochholdinger and Tuberosa, 2009), which we used here to investigate maize root hair formation induced changes in rhizosphere *acdS* community composition. *Zea mays* root hair defective mutant *rth3* and the corresponding B73 wildtype (Wen and Schnable, 1994; Hochholdinger et al., 2008) seeds were propagated at the experimental station Endenich of the Faculty of Agriculture of the University of Bonn. The monogenic mutant *rth3* is transposon induced and shows normal root hair initiation but disturbed elongation (Hochholdinger et al., 2008).

### Experimental Design and Conduction of the Experiment

The basic design of the soil column experiment consisted of three factors and six replications, referring to six planted soil columns per treatment. The experimental factors included the substrate represented by loam (L) and sand (S) and the maize genotype represented by wild type (WT) and *rth3*. This resulted in 24 columns. The third experimental factor did not affect column replicate number; it was the depth of the column at three equally spaced layers (D1, D2, and D3) (Supplementary Figure 1). Substrates L and S were sieved to a particle size of ≤1 mm and fertilized with 50 mg N (NH_4_NO_3_), 50 mg K (K_2_SO_4_), 25 mg Mg (MgCl_2_ × 6 H_2_O), and 40 mg P (CaHPO_4_) per kg substrate dry mass for loam, but with 100 mg N (NH_4_NO_3_), 100 mg K (K_2_SO_4_), 50 mg Mg (MgCl_2_ × 6 H_2_O), 80 mg P (CaHPO_4_), 100 mg CaSO_4_ × 2 H_2_O, 3.25 mg MnSO_4_ × H_2_O, 0.79 mg Zn(NO_3_)_2_ × 4 H_2_O, 0.5 mg CuSO_4_ × 5 H_2_O, 0.17 mg H_3_BO_3_ and 3.25 mg FeEDTA for sand. Substrates were filled into acrylic columns (25 cm height, 7 cm inner diameter, 0.5 cm wall thickness) with a bulk density of 1.26 g cm^–3^. Maize seeds were surface-sterilized using 10 min 10% H_2_O_2_, 5 min H_2_O, followed by soaking in saturated CaSO_4_ for 3 h. The surface sterilized seeds were sown 1 cm below the substrate surface and covered with coarse gravel to reduce evaporation. Aluminum foil was placed around the columns to prevent algal growth, and irrigation was implemented from top and bottom to maintain a water content of 22% (v/v) throughout the growth period. The plants were grown for 22 days in a growth chamber with the following settings: 12 h light period at 350 μmol m^–2^ s^–1^ photosynthetically active radiation, 22°C/18°C day/night temperature and 65% humidity.

Cultivation was terminated at 3 weeks age of the plants as the growth of the maize plants is restricted in soil columns, which might affect root distribution and physiology (Poorter et al., 2012). Shoot height and leaf number were measured at harvest. Plant growth was not affected by substrate, but *rth3* plant height and water consumption were lower than that of WT (Supplementary Figure 1). The harvest was implemented at three depths by horizontal cuts through the whole column. Maize roots were collected from soil slices of approximately 1.6 cm thickness at 4.5–6.1 cm (depth 1, D1), 9.0–10.6 cm (depth 2, D2), and 13.5–15.1 cm (depth 3, D3) below the soil surface (Supplementary Figure 1). Roots with adhering soil were picked up with tweezers. To obtain the material for the analyses of the rhizosphere microbiome, these roots were gently shaken and briefly submerged and shaken in sterile 0.3% NaCl [1:10 (w/v) dilution, i.e., 1 g root fresh mass + 9 mL NaCl]. The rhizosphere suspension was centrifuged at 5,000 × *g* for 30 min at 4°C and the rhizosphere pellets were stored at −20°C before DNA extraction.

### *acdS* Illumina Library Preparations

Microbial composition was analyzed for six rhizosphere samples corresponding to six individual soil columns per treatment and sampling depth. Total DNA was extracted from the rhizosphere pellets (about 500 mg fresh mass) using FastDNA Spin Kit and Geneclean Spin Kit for soil following the manufacturer’s instructions (MP Biomedicals, United States). Partial *acdS* gene was amplified in duplicate using the primers *acdSF5* and *acdSR8* (Bouffaud et al., 2018). Primers were equipped with Illumina adapter sequences (Nextera XT Index Kit, Illumina, San Diego, CA, United States). To obtain error-poor amplifications, PCR was performed using Kapa Hifi HotStart ReadyMix (Kapa Hifi HotStart, KAPA-Biosystems, Wilmington, MA, United States). The PCR was performed in a S1000 Thermal Cycler (Bio-Rad Laboratories, Hercules, CA, United States) and the cycling conditions were 30 cycles of 65°C for 10 s, 72°C for 10 s. PCR products were purified using AMPure XP beads and, to assign the sequences to the respective samples, an index PCR was performed using the Illumina Nextera XT Index Kit and Kapa Hifi HotStart ReadyMix (KAPA Biosystems, Wilmington, MA, United States). The PCR was performed in a S1000 Thermal Cycler (Bio-Rad Laboratories, Hercules, CA, United States) and the cycling conditions were 8 cycles of 95°C for 30 s, 55°C for 30 s, and 72°C for 30 s. The index PCR products were purified with AMPure XP beads and the PCR products were quantified with Quant-iT PicoGreen dsDNA Assay Kit (Invitrogen, Life Technologies, Carlsbad, CA, United States) following manufacturer’s instructions and using Cary Eclipse Fluorescence Spectrophotometer (Agilent Technologies, Santa Clara, CA, United States) with an excitation wavelength of 480 nm and an emission wavelength of 520 nm. The size and the quality of the pooled PCR products were analyzed using Agilent 2100 Bioanalyzer (Agilent). The *acdS* libraries were sequenced on an Illumina MiSeq system to 2 × 150 nucleotide lengths. Project design is described at NCBI under the Bioproject PRJNA666771, and raw sequences are placed in Short Reads Archive under the accessions SRX9227754-SRX9227812.

### Sequencing Processing and Statistics

Only reads with the adequate primers were kept. The obtained sequences were analyzed using dadasnake (v. 0.7; Weißbecker et al., 2020^1^) which uses the open source program R’s (v. 3.6.1; R Core Team 2017) DADA2 package (Callahan et al., 2016). *acdS* gene amplicon reads were cut until the lowest possible quality of a base was 11 and truncated to 100 and 90 nt for the forward and reverse reads overall length. The overall maximum expected error was constrained to 0.7. Read pairs were merged with zero mismatches, and exact sequence variants were determined to be used as ASVs (Amplicon Sequence Variants). Chimeric reads were removed using the DADA2 “consensus” algorithm (Callahan et al., 2016). The *acdS* sequences were aligned against an *acdS* in-house database extracted from FunGene (Fish et al., 2013) using BLASTn (v. 2.7.1) according to Bouffaud et al. (2018), and the ASVs not assigned taxonomically using BLASTn were removed. Two samples which were represented by less reads after quality control, ASV generation, chimera removal and taxonomic assignment steps than others were excluded from further analyses. These were one sample for loam WT maize at sampling depth D2 and one sample for sand rth3 maize at sampling depth D3 (Supplementary Table 1 and Supplementary Figure 2). After this, all treatments were represented by five replicates except for loam WT maize at sampling depth D2 and sand rth3 maize at sampling depth D3, for which only four replicates were further analyzed. A phylogenetic tree was constructed from a multiple alignment of the ASVs using mafft (v7.455; Katoh et al., 2002) by running fasttreeMP (v. 2.1.10) with the gamma option. Tips with more than 98% sequence identity were collapsed for visualization and renamed with the most abundant ASV or a random reference (if there were no ASVs in the set of collapsed tips). The tree was visualized in iroki (Moore et al., 2020). Analysis scripts are accessible at https://github.com/a-h-b/maize-acdS.

All statistical analyses and visualizations were performed in R. ASV patterns were cross-compared with permutational multivariate analysis of variance (PERMANOVA) using the “vegan” package. Since strongly skewed datasets were identified, apart from using ANOVA and Tukey’s tests, Kruskal–Wallis test followed by *post hoc* Dunn-Bonferroni test were carried out with “PMCMRplus.” The effect of watering during the conditioning phase was tested for each *acdS* genus, comparing the farming system and plant factor independently, using DESeq2 (Love et al., 2014) and visualized using pheatmap (Kolde and Kolde, 2015; Kolde, 2019).

## Results

### Estimation of *acdS* Species Richness and Diversity

**FIGURE 1:**
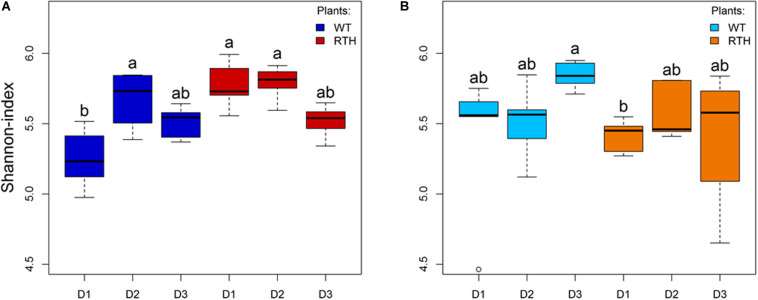
Shannon indices of the *acdS* sequences. Loam **(A)** and sand **(B)** substrate data of wild type (WT) and *rth3* (RTH) maize seedlings from three depths, with uppermost sampling depth D1, middle depth D2, and lowest depth D3, were examined by Kruskal–Wallis test followed by *post hoc* Dunn-Bonferroni test. Different letters indicate significant differences (*p* < 0.05).

Rhizosphere DNA was subjected to *acdS* amplicon sequencing. The total number of 3,838,082 reads clustered into 4,083 ASVs. The sequences were rarefied to the smallest read number per sample, 39,398 reads. At this level, all rarefaction curves reached their plateau, showing that this normalization was not at the expense of a satisfying sequencing depth (Supplementary Figure 2). Substrate and maize genotype affected the richness of observed ASVs. Higher average numbers of ASVs (ANOVA, *P* = 0.03) were detected in loam than in sand, and in loam, the average number of ASVs for *rth3* was higher than for WT maize seedlings (*P* = 0.0005). The numbers of ASVs neither varied significantly between the root hair genotypes in sand nor between the depths where the samples were taken (Supplementary Table 2). Shannon index, a diversity measure combining the number of taxa and their evenness, was similar for loam (Figure 1A) and sand (Figure 1B) and for the three depths. Nevertheless, the Shannon index of WT in D1 was lower than that of *rth3* in D1 and D2 in loam, and the index of WT in D3 was higher than rth3 in D1 in sand. Variation in Shannon indices was particularly high for *rth3* in D3.

### Factors Shaping *acdS* Carrying Community Composition

**FIGURE 2:**
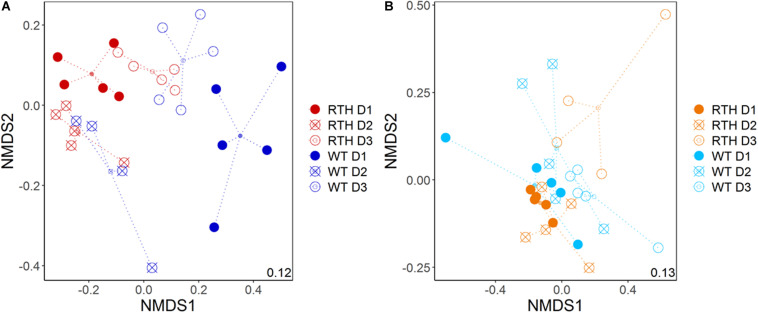
Non-metric dimensional scaling (NMDS) plot of *acdS* sequence distribution for maize rhizospheres in loam **(A)** and sand **(B)**. WT, wild type; RTH, *rth3* mutant; D1, uppermost sampling depth; D2, middle depth; D3, lowest depth.

Soil texture was the main factor influencing the composition of *acdS* carrying (*acdS+)* bacterial communities, followed by the depth, and the effect of the maize root hair genotype according to a PERMANOVA analysis (Table 1 and Supplementary Figure 3). The *acdS+* communities from the loam and sand substrates were separately analyzed in order to visualize the plant genotype and depth effects (Figure 2 and Table 1). In both substrates, the depth had a major impact on the *acdS+* community. The impact of genotype was stronger in loam than in sand, and particularly strong in loam at the uppermost depth D1.

**TABLE 1:** PERMANOVA of the 1-aminocyclopropane-1-carboxylate deaminase gene (*acdS*) community composition.

**Both soil textures**		

	*R*^2^	*P*
Soil	0.354	0.001
Genotype	0.020	0.032
Depth	0.071	0.001
Soil:Genotype	0.020	0.051
Soil:Depth	0.067	0.001
Genotype:Depth	0.029	0.074
Soil:Genotype:Depth	0.029	0.100
Residuals	0.410	

**Loam**		

	***R*^2^**	***P***

Genotype	0.072	0.001
Depth_L > Depth	0.299	0.001
Genotype:Depth	0.093	0.003
Residuals	0.537	

**Sand**		

	***R*^2^**	***P***

Genotype	0.054	0.024
Depths > Depth	0.140	0.001
Genotype:Depths > Genotype:Depth	0.087	0.034
Residuals	0.719	

*The ASV composition was considered for all treatments, to determine the effects and relative importance of substrate (loam and sand), plant genotype (wild type and rth3 mutant maize), and sampling depth. Since the soil:depth interaction was significant, the relative importance of plant genotype and sampling depth are also given for each substrate. Note that the genotype has a stronger impact in loam than in sand.*

### Taxonomic Affiliations of the *acdS* Sequences and Factors Affecting Their Distribution

**FIGURE 3:**
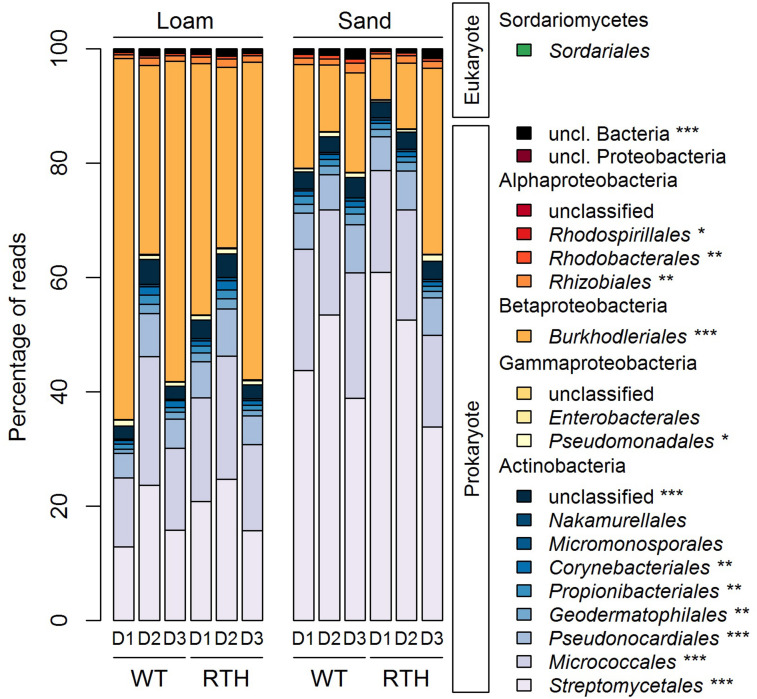
Relative abundance of *acdS* sequences in the different orders in columns with loam (L) and sand (S) of wild type B73 (WT) and *rth3* (RTH) maize rhizosphere in three sampling depths (D1, D2, and D3). Different relative abundances of *acdS* sequences at the order level according to Kruskal–Wallis and *post hoc* Dunn-Bonferroni test are marked by asterisks (***P < 0.001, **P < 0.01, *P < 0.05). Statistical differences indicated in this figure are related to substrate, plant genotype, and/or sampling depth, and they are described in Supplementary Table 2.

**FIGURE 4:**
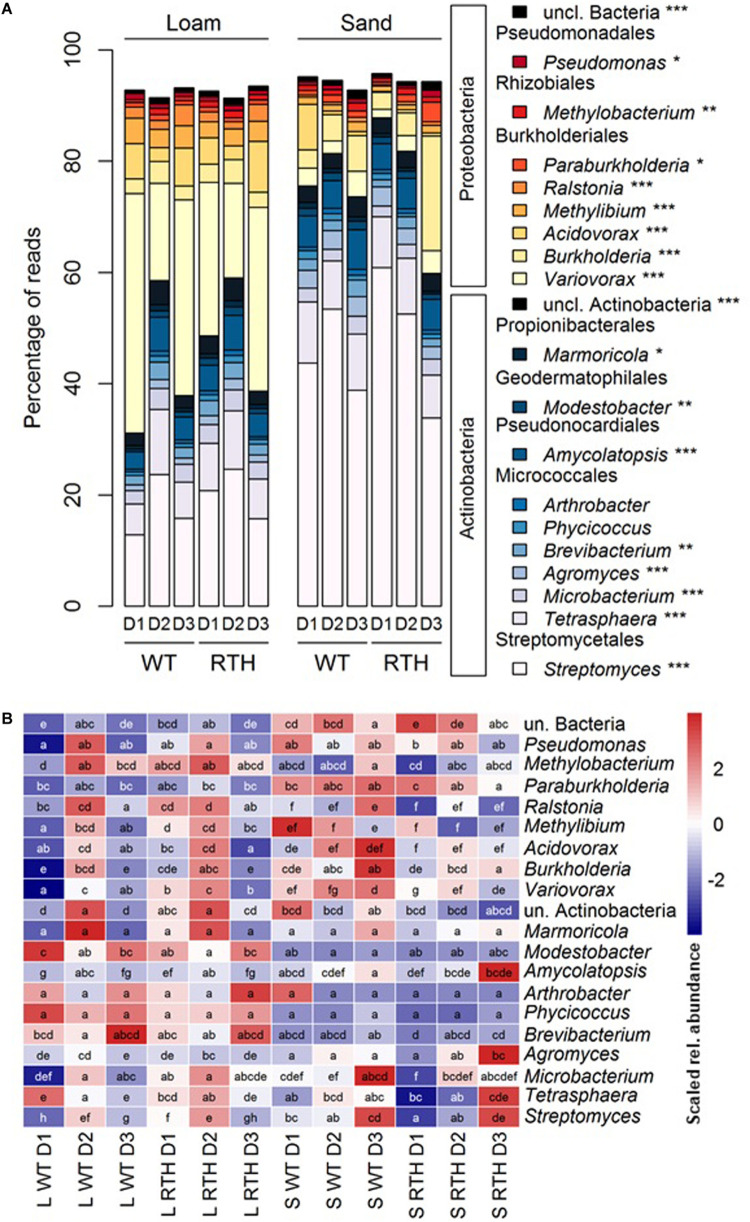
Effects of substrate, maize genotype, and sampling depth on the 20 most-abundant genera according to *acdS* analysis. Relative abundances of the 20 most abundant genera in maize rhizosphere in loam and sand with wild type B73 (WT) and *rth3* (RTH) maize and three sampling depths (D1, D2, and D3). **(A)** Distribution of the genera in Actinobacteria and Proteobacteria. Asterisks mark differential distribution across the treatments according to Kruskal–Wallis test followed by Dunn *post hoc* test. **(B)** Statistics of the relative abundances across treatments. Red color indicates high relative mean number of sequences and blue color low relative mean number of sequences of a genus level. Different letters indicate significant differences according to Kruskal–Wallis test followed by Dunn *post hoc* test.

The *acdS* amplicons were largely associated with two phyla, Actinobacteria and Proteobacteria. As a demonstration of the phylogenetic classification of the *acdS* amplicons, we determined a tree (Supplementary Figure 4) using the ASVs and published reference sequences. There were two major clusters in the phylogenetic tree, according to the main represented phyla Actinobacteria and Proteobacteria. At the order level, Proteobacteria corresponded mainly to Burkholderiales, whereas the Actinobacteria were dominated by the Streptomycetales and Micrococcales, and at a lesser extent by the Pseudonocardiales (Figure 3; statistical differences related to substrate, plant genotype, and/or sampling depth in Supplementary Table 3). Sequences from eukaryotic microorganisms of the fungal order Sordariales were also detected. The *acdS* sequences from the 20 most abundant genera in maize rhizospheres represented 91% of the total number of reads (Figure 4A), and the dominant distribution of Actinobacteria and Proteobacteria ASVs was preserved. Except for the genera *Pseudomonas* and *Methylobacterium*, the other six genera from the Proteobacteria corresponded to the order Burkholderiales from Betaproteobacteria, and among the Actinobacteria, the ten genera were distributed in five different orders. *Variovorax, Acidovorax*, and *Burkholderia* of Burkholderiales, as well as *Streptomyces* of Streptomycetales and *Tetrasphaera* of Micrococcales were among the most numerous *acdS+* genera.

As indicated by the PERMANOVA, the substrate had a strong effect on *acdS* sequence composition. Compared to loam, sand showed lower relative abundance of *acdS* carrying Proteobacteria (34–72% vs. 8.6–53.9%), and higher abundance of Actinobacteria (28–65% vs. 46–92%), caused predominantly by lower relative abundance of Burkholderiales but higher relative abundance of Streptomycetales *acdS* sequences in sand (Figure 3; statistical differences at order level are described in Supplementary Table 3). Abundance of *acdS* reads from the genera *Streptomyces* and *Agromyces* was higher in the sand samples, whereas in loam *Variovorax, Acidovorax, Ralstonia*, and *Methylibium* were more dominant (Figure 4).

The phyla displayed marked differences in their relative abundances between the three depths of the column (descending order D1, D2, and D3). Higher relative abundances of Actinobacteria sequences were evident in the D2 than in D1 or D3, in particular in loam. Differences across the treatments were found, e.g., for Micrococcales, Propionibacteriales, Pseudonocardiales, and unclassified Actinobacteria with particularly high abundance in D2 in loam. By contrast, the *acdS* sequences from Burkholderiales displayed lower relative abundances in D2 than D1 or D3 in loam (Figure 3 and Supplementary Table 3). Depth also influenced the abundance of *acdS* from several genera (Figure 4). In loam, the effect of depth was evident on the level of genera *Streptomyces, unclassified Actinobacteriaceae, Tetrasphaera*, and *Amycolatopsis acdS*, which were more prevalent in D2. In sand, *Paraburkholderia, Burkholderia*, and *Variovorax acdS* were more abundant in D3.

Finally, maize genotype affected some *acdS+* phyla as well, particularly in the uppermost layer D1. Regardless of substrate, Burkholderia *acdS* at order level and *Variovorax acdS* at genus level were more abundant in WT rhizosphere. Furthermore, for loam we observed higher abundance of and higher abundance of *Methylibium acdS* in WT rhizosphere. By contrast, Streptomycetales *acdS* at order level and *Streptomyces acdS* at genus level were more abundant in the *rth3* rhizosphere regardless of substrate, and in loam, same pattern occurred for *Microbacterium* and *Modestobacter acdS*.

## Discussion

### General Differences in *acdS+* Community Structure Across Soil Textures

Glick (2014) argued that the more precisely the drivers of PGPR distribution and activity are researched, the more likely it is that novel means can be developed to support plant growth in the changing environment. Contributing to this aim we investigated *acdS* carrying *(acdS+)* microbial community composition in maize rhizosphere in a multifactorial design. We observed that the main drivers in descending order of impact were substrate, depth and maize genotype. These observations essentially confirmed our first hypothesis, and suggest that in the frame of functional redundancy among ACC deaminase phyla, maize rhizosphere selects rapidly a suitable *acdS+* bacterial community that maintains ACC deaminase activity in the rhizosphere.

### *acdS+* Communities of Maize Rhizospheres

The maize cultivar of this study, B73, is a cultivar of the Corn Belt dent genetic group of maize (Labate et al., 2003). For Mo17 and FV252, which are representatives of the same genetic group, Bouffaud et al. (2018) found a similar phylogenetic distribution of the 20 most abundant *acdS+* bacterial genera to the current study: All genera correspond to Proteobacteria and Actinobacteria, contributing over 90% of the sequences, including *acdS+* bacterial genera *Burkholderia, Streptomyces, Acidovorax, Variovorax*, and *Methylibium*. Of these, members of *Streptomyces* have been characterized as efficient biocontrol agents through production of antimicrobials, but also as plant growth and health stimulators (Merriman et al., 1974; Xiao et al., 2002; Schrey and Tarkka, 2008). ACC deminase activity plays a crucial role in this process. For instance, inoculation with *Streptomyces* sp. GMKU 336 stimulates plant shoot and root growth, enhances chlorophyll levels but suppresses formation of ethylene and reactive oxygen species - effects which are absent during inoculation with an ACC deaminase-deficient strain (Jaemsaeng et al., 2018). Similarly, an ACC deaminase overproducing strain of *Streptomyces venezuelae* ATCC 10712 stimulated shoot growth and elongation more strongly than the wild type strain (Yoolong et al., 2019).

### Substrate: The Main Driver of the *acdS+* Bacterial Community

The *acdS+* substrate responder genera in this experiment, i.e., those with a differences in their relative abundances between loam and sand, included several genera with substrate-dependent relative abundance patterns at the 6 leaves stage of maize identified by Bouffaud (Renoud et al., 2020). Growth stage of the present experiment (BBCH14 stadium) corresponds to the 6 leaves stage. This confirms our second hypothesis on the relation between the substrate and different types of field soil used by Renoud et al. (2020). Out of the 20 most abundant *acdS+* genera in this study, the relative abundances of *Burkholderia, Ralstonia*, and *Variovorax* were altered by substrate, and they belonged to the genera with differences in distribution between three field sites representing luvisol, fluvic cambisol and calcisol substrates that were studied by Renoud et al. (2020). Of note, *acdS* gene is not only confined to PGPR, since some members of these genera include plant pathogens, e.g., *R. solanacearum* of *Ralstonia* and *P. syringae* of *Pseudomonas*, and they both possess the *acdS* gene. Other members of *Pseudomonas* are reported to exhibit a wide range of plant growth-promoting traits. This comprises ACC deaminase activity, which is widespread also among the cultivated members of *Pseudomonas* for maize rhizosphere (Vacheron et al., 2016b). *acdS+ Pseudomonas* isolates promote maize growth (Shaharoona et al., 2006) and may reduce stress-induced ethylene levels of maize. For instance under salt stress, *acdS+ Pseudomonas fluorescens* strain enhanced N, P, and K uptake and increased maize biomass and grain yield over the control (Nadeem et al., 2009). Substrate may not only modify the abundance but it may also affect the relative distribution patterns of *acdS+ Pseudomonas* strains between rhizosphere and bulk soil. Vacheron et al. (2016b) observed that the proportion of *acdS+ Pseudomonas* can be higher, lower, or unchanged in maize rhizosphere than bulk soil, depending on substrate. It has also been described that the relative expression of the *Pseudomonas acdS* gene is modulated by maize genotype, and *Pseudomonas acdS-* mutant analyses suggested that the ACC deaminase activity of *P. fluorescens* contributes to either root branching or stress alleviation, depending on maize genotype (Vacheron et al., 2016a).

At the level of 16S rRNA gene amplicon sequencing, it is established that soil physics and chemistry are often the strongest factors in shaping the total rhizospheric bacterial community structure and function, in particular soil particle size and pore distribution, moisture, pH and nutrient availability (Berg and Smalla, 2009; Fierer, 2017; Wang et al., 2019). In this work, we compared plants grown in loam (haplic phaeozem) and sand (ratio between quartz sand and haplic phaeozem 83.3 to 16.7%), the substrates of the central experiments of the priority program Rhizosphere Spatiotemporal Organization – PP 2089 (Vetterlein et al., 2020a). To achieve similar plant nutrition and achieve similar water distribution in the soil columns, nutrient differences were equilibrated by the different fertilizer regimes for the two substrates, and watering was carried out from the top and the bottom of the columns (Vetterlein et al., 2020b). The fact that the impact of the substrate on the *acdS+* community was highly significant implies that soil texture has a major impact on rhizosphere properties and plant-microbe interactions. Feedback processes between the plant and the microorganisms should not be neglected, since soil texture also influences root architecture and root physiology (Rich and Watt, 2013; Rogers et al., 2016; D. V., Eva Lippold, Maxime Phalempin, Steffen Schlüter, unpublished data). Fischer et al. (2010) observed in a similar approach than the one used here, i.e., fewer sorption sites in the mixture than in the original soil, that higher concentrations of leached substances and increased maize root exudation was observed in the soil-sand mixture. In the maize system, higher maize secondary metabolism (phenylpropanoid and terpenoid metabolism) and plant immunity (pathogenesis related protein genes) gene expression was observed in sand than in loam, as well as increased water channel expression (M. G., unpublished data). This could indicate that the plant exerts a stronger selective influence on the *acdS+* community in sand than in loam.

### The Relation Between Depth and *acdS+* Community Composition

Community composition of *acdS+* bacteria was strongly affected by the depth of the soil column, with increased relative abundances of the members of *acdS+* Burkholderiales (*Paraburkholderia*, *Burkholderia*, and *Variovorax)* in D3, but *acdS+* Actinobacteria (*Streptomyces, Tetrasphaera*, and *Amycolatopsis*) in D2. Of these, a widespread ACC deaminase activity in *Burkholderia* species has been indicated by a cultivation based analysis. From a *Burkholderia* collection, 18 out of 20 species (90%) exhibited ACC deaminase activity (Onofre-Lemus et al., 2009). Enhancement of growth and salt tolerance of rice seedlings by ACC deaminase-producing *Burkholderia* strain was not supported by ACC deaminase mutant that was not able to reduce stress ethylene, confirming that the plant beneficial impact was associated with a reduction in ethylene level (Sarkar et al., 2018). And, Huang et al. (2017) reported that among rhizosphere bacterial isolates of plants subjected to water limitation, *Burkholderia* isolate was among the two most powerful PGPR. That particular *Burkholderia* strain exhibited ACC deaminase activity and promoted drought tolerance by decreasing plant ethylene levels. Preliminary results from the analysis of the root systems from a comparable experiment (D. V., Eva Lippold, Maxime Phalempin, Steffen Schlüter, unpublished) indicates that roots grow fast through the substrate and that the density of young roots (0–7 days old) in the lower section of the columns is higher than on the top at this growth stage. This indicates that changes in *acdS+* taxa distribution between different depths could be, at least in part, explained by changes in the distribution of young, active roots. Resource quality and distribution might play a role in the differences between the enrichment patterns of Burkholderiales and actinobacteria, since whereas the first have often been affiliated as copiotrophs, highly responsive to easily degradable carbon and other resources upon availability (Padmanabhan et al., 2003; Fierer et al., 2007; reviewed in Ho et al., 2017), actinomycetes commonly contribute to decomposition through active breakdown of plant biomass (Lewin et al., 2016). To test this hypothesis, rhizodeposition at different depths of the column should be investigated.

### The *roothairless3* Mutant

Despite their importance for plant growth (Bates and Lynch, 2000) and nutrient acquisition (Zhu et al., 2010), information on the role of the root hairs in rhizosphere microbiome assembly is limited. Root hairs extend into the rhizosphere and alter its spatial dimensions. They take up nutrients and modulate resource distribution in the rhizosphere, and thus generate micro-niches that could enrich some but deplete other members of the rhizosphere microbiota (Pascale et al., 2020). The genotype effects on the maize rhizosphere microbiota included an increase in the relative abundances of *acdS+* Actinomycetes; *Streptomyces* in both substrates, but *Microbacterium* and *Modestobacter* only in loam in *rth3*, compared to an increase of the Burkholderiales *acdS+* genera in WT, *Variovorax* in both substrates, but *Methylibium* only in loam. This observation confirmed our third hypothesis, stating that the effect of plant genotype differs between loam and sand. In this respect, it is in accordance with the rhizosphere 16S rRNA gene amplicon sequencing results of WT and root hair mutant barley (Robertson-Albertyn et al., 2017). Interestingly, while maize *acdS+* bacteria predominantly differed in their responses to *rth3* in loam, the barley total bacterial community composition responded stronger to plant genotype in a substrate with lower organic matter content (Robertson-Albertyn et al., 2017). Higher richness of *acdS+* ASVs of *rth3* maize than the WT was also in contrast to barley total bacterial OTU richness, which showed no obvious differences between WT than root hair mutant plants (Robertson-Albertyn et al., 2017). Ultimately, the comparison between maize *acdS* and total bacterial community studies (K. S. and B. Y., unpublished data) will determine the correspondence between the *acdS* and the 16S rRNA gene-based community composition patterns, and the relatedness between the maize and barley results. The current study indicates that the importance of the *rth3* mutation, or the association between elongated root hairs and the structure of the rhizosphere microbiome, is higher in the upper (D1) than lower (D2 and D3) area of the soil column. Our preliminary results (Nina Naderi, Sophie Wachter, M. T. T., and D. V., unpublished data) suggest that the general occurrence of root hairs is similar in loam and sand in the column system, but we have not yet investigated if their density or elongation are affected by depth.

### Perspectives

Following the findings in this study, we anticipate that the spatial distribution of the *acdS* microorganisms is likely a vital feature of bacterium-plant interactions. Our recently conducted experiment (Ganther et al., 2020) confirmed that X-ray computed tomography is compatible with a subsequent microbiome analysis, facilitating further research on the spatial distribution of the *acdS* microorganisms. Future work should also target the temporal, plant age associated variation in *acdS* abundance and population structure.

## Data Availability Statement

The datasets presented in this study can be found in online repositories. The names of the repository/repositories and accession number(s) can be found below: https://www.ncbi.nlm.nih.gov/, Bioproject PRJNA666771.

## Author Contributions

MG, MT, and M-LB designed the experiment. LG performed and analyzed the experiment with input from AH-B, M-LB, and MT. BY prepared rhizosphere DNA. AH-B provided assistance for the bioinformatics pipeline and data analysis. LG, M-LB, and MT wrote the manuscript with contributions from all authors. All authors contributed to the article and approved the submitted version.

## Conflict of Interest

The authors declare that the research was conducted in the absence of any commercial or financial relationships that could be construed as a potential conflict of interest.

## Footnotes

1 https://github.com/a-h-b/dadasnake

## Supplementary Material

Supplementary Figure 1 Experimental design, maize growth, and water consumption.

Supplementary Figure 2 Rarefaction curves for partial *acdS* sequences.

Supplementary Figure 3 Distribution of the *acdS* sequences is the most strongly affected by substrate.

Supplementary Figure 4 Tree of amplicon sequence variants and reference sequences.

Supplementary Table 1 Summary of substrate characteristics for loam and sand.

Supplementary Table 2 Frequency of *acdS* amplicon sequencing variants in maize rhizosphere.

Supplementary Table 3 Relative abundances of *acdS* sequences affiliated to the order level across all treatments.
