# Supplementary material for: Soil Texture, Sampling Depth and Root Hairs Shape the Structure of ACC Deaminase Bacterial Community Composition in Maize Rhizosphere
Source: Front Microbiol. 2021 Feb 4;12:616828. doi: 10.3389/fmicb.2021.616828 (PMC7891401; doi:10.3389/fmicb.2021.616828)

Supplementary Material

Supplementary Table S1. Summary of substrate characteristics for loam and sand.

Supplementary Table S2. Frequency of *acdS* amplicon sequencing variants in maize rhizosphere.

Supplementary Table S3. Relative abundances of *acdS* sequences affiliated to the order level across all treatments.

Supplementary Figure S1. Experimental design, maize growth and water consumption.

Supplementary Figure S2. Rarefaction curves for partial *acdS* sequences.

Supplementary Figure S3. Distribution of the *acdS* sequences is the most strongly affected by substrate.

Supplementary Figure S4. Tree of amplicon sequence variants and reference sequences.

Supplementary Table S1. Summary of substrate characteristics for loam and sand. Initial values for loam, quartz sand and their mixture sand (16.7% loam, 83.3% quartz sand) are provided. The loam was obtained by sieving and homogenising haplic Phaeozem excavated from 0-50 cm depth at Schladebach (51°18’31.41” N; 12°6’16.31” E), and the quartz sand WF33 was obtained from Quarzwerke GmbH. *Values for sand were calculated based on results of loam and quartz sand at a 16.7:83.3 mixing ratio

**Substrate**	**Bulk density** [g cm^{-3}]	**pH ($CaCl_2$)**	**Carbonate** [g kg^{-1}]	**Sand** [%]	**Silt** [%]	**Clay** [%]	**CEC** [$mmol_c$ kg^{-1}]	**C_{org}** [%]	**N_t** [%]	**P plant available** [mg kg^{-1}]	**K plant available** [mg kg^{-1}]	**Fe_o** [g kg^{-1}]
Loam	1.26	6.21	<1	33.2	47.7	19.1	76.1	0.84	0.084	33.41	26.67	1.32
Sand*	1.47	6.25	<1	88.6	8.1	3.3	13	0.14	0.014	5.67	5.44	0.22
Quartz sand		5.8	<1	99.8	0.1	0.1	0.33	0	0	0.11	1.19	0

Supplementary Table S2. Frequency of *acdS* amplicon sequencing variants in maize rhizosphere. Abbreviations: loam (L) and sand (S), wild type B73 (WT) and *rth3* (RTH), descending sampling depths (D1, D2 and D3), and samples (A-E).

**Sample**	**D1**	**D2**	**D3**	**Sample**	**D1**	**D2**	**D3**
L WT A	366	433	522	L RTH A	503	505	547
L WT B	378	322	411	L RTH B	542	516	521
L WT C	451		405	L RTH C	531	536	445
L WT D	392	542	447	L RTH D	595	412	429
L WT E	381	501	450	L RTH E	586	571	472
Average	393.6	449.5	447	Average	551.4	508	482.8
Std error	14.94	48.08	20.85	Std error	17.24	26.50	22.38
**Sample**	**D1**	**D2**	**D3**	**Sample**	**D1**	**D2**	**D3**
S WT A	456	365	583	S RTH A	462	477	405
S WT B	411	381	463	S RTH B	407	419	511
S WT C	500	410	481	S RTH C	408	389	365
S WT D	242	539	461	S RTH D	382	396	376
S WT E	371	425	516	S RTH E	367	527	
Average	396	424	500.8	Average	405.2	441.6	414.25
Std error	44.15	34.23	22.80	Std error	16.18	26.37	33.34

Supplementary Table S3. Relative abundances of *acdS* sequences affiliated to the order level across all treatments. Abundance of reads in the different orders in columns with loam (L) and sand (S) in three sampling depths (D1, D2 and D3) of wild type B73 (WT) and *rth3* (RTH) maize rhizospheres. Different letters indicate different relative abundances of *acdS* sequences affiliated to an order according to Kruskal-Wallis and post-hoc Dunn-Bonferroni test (P<0.05). uncl., unclassified.

**Treatment & depth**	**Sordariales**	**uncl. Bacteria**	**uncl. Proteobacteria**	**uncl. Alphaproteobacteria**	**Rhodospirillales**	**Rhodobacterales**	**Rhizobiales**	**Burkholderiales**	**uncl. Gammaproteobacteria**	**Enterobacterales**	**Pseudomonadales**	**uncl. Actinobacteria**	**Nakamurellales**	**Micromonosporales**	**Corynebacteriales**	**Propionibacteriales**	**Geodermatophilales**	**Pseudonocardiales**	**Micrococcales**	**Streptomycetales**
**L RTH D1**	a	bcd	a	a	b	bcd	abc	b	a	a	ab	abc	a	a	bcde	abcd	ab	cd	bcd	f
**L RTH D2**	a	ab	a	a	b	cd	a	c	a	a	a	a	a	a	a	a	a	a	ab	e
**L RTH D3**	a	de	a	a	b	d	abc	a	a	a	ab	cd	a	a	de	bcd	cd	de	de	gh
**L WT D1**	a	e	a	a	b	d	c	a	a	a	a	d	a	a	e	cd	d	e	e	h
**L WT D2**	a	abc	a	a	b	bcd	ab	bc	a	a	ab	a	a	a	ab	a	abc	abc	a	ef
**L WT D3**	a	de	a	a	b	d	bc	a	a	a	ab	d	a	a	abc	d	abcd	de	de	g
**S RTH D1**	a	e	a	a	b	d	bc	f	a	a	b	bcd	a	a	e	abcd	abcd	cde	bcd	a
**S RTH D2**	a	de	a	a	b	abcd	ab	ef	a	a	ab	bcd	a	a	cde	abcd	abc	abc	abc	ab
**S RTH D3**	a	abc	a	a	ab	abcd	abc	bc	a	a	ab	abcd	a	a	cde	abcd	bcd	cd	cde	de
**S WT D1**	a	cd	a	a	ab	ab	abc	de	a	a	ab	bcd	a	a	cde	ab	abc	bcd	abc	bc
**S WT D2**	a	bcd	a	a	a	abc	abc	e	a	a	ab	bcd	a	a	cde	abcd	ab	cde	abcd	ab
**S WT D3**	a	a	a	a	ab	a	a	d	a	a	ab	ab	a	a	abcd	abc	ab	ab	ab	cd
**K.W. test**	**n.s.**	***	**n.s.**	**n.s.**	*	**	**	***	**n.s.**	**n.s.**	*	***	**n.s.**	**n.s.**	**	**	**	***	***	***

Supplementary Figure S1. Experimental design, maize growth and water consumption. Wild type and *rth3* mutant maize were grown from seeds for three weeks in loam or sand and 1.6 cm sections of the soil columns were harvested from three sampling depths (D1 D2 and D3) by cutting with a blade. Shoot height and leaf number were evaluated before harvest, and water use daily. Values correspond to the average value of six replicates referring to six columns. Letters show the statistical differences between treatments, for leaf number according to Kruskal-Wallis test followed by Dunn post hoc test and for height and water consumption according to ANOVA and Tukey test.

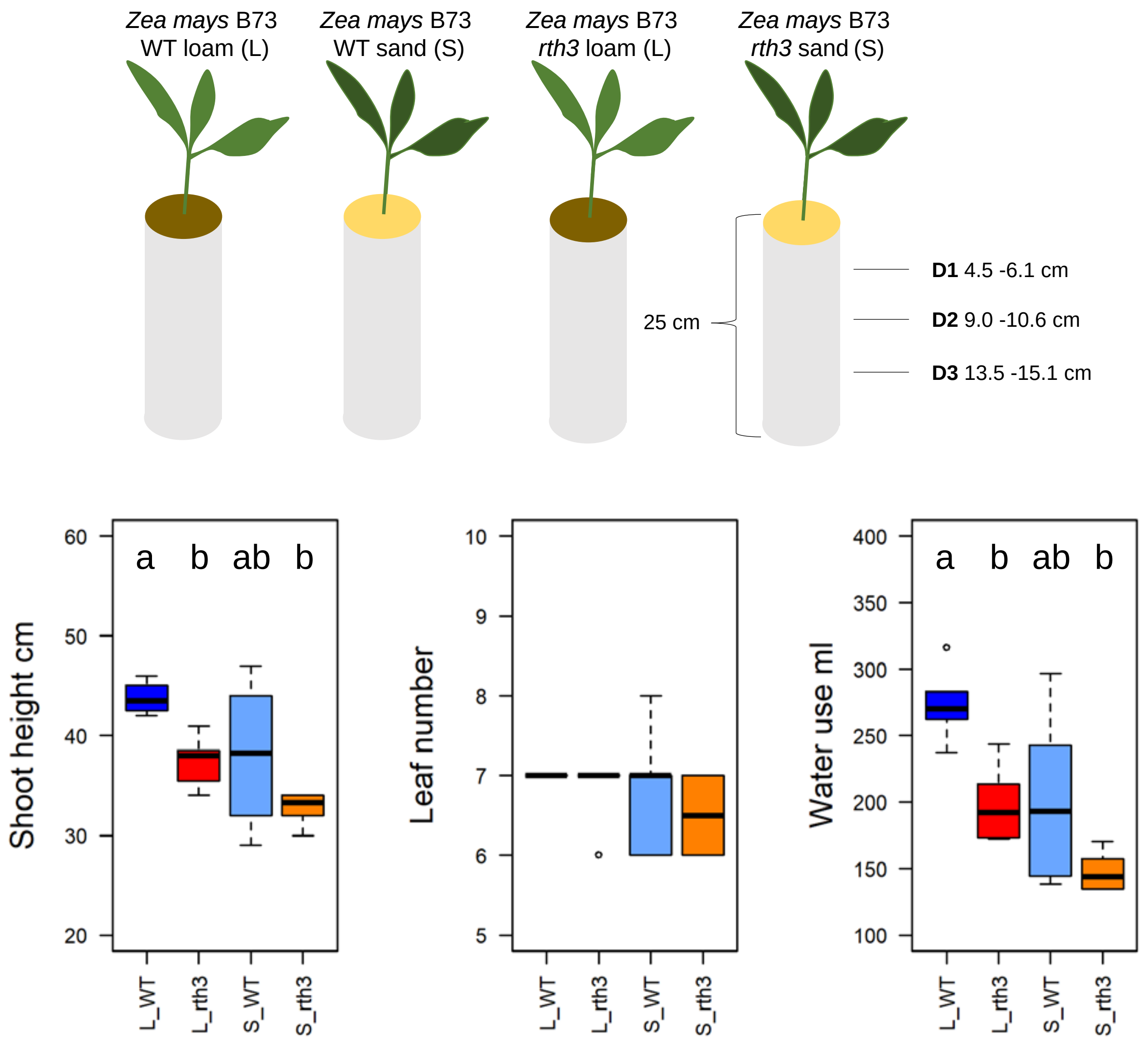

Supplementary Figure S2. Rarefaction curves for partial for 1-aminocyclopropane-1-carboxylate deaminase (*acdS*) sequences. Shown are rarefaction curves of all samples. Blue marks rarefaction curves of partial acdS genes of wild type (WT) and orange rth3 (RTH) maize seedlings, dark colours mark loam (L) and light colours sand (S), and the sampling depth D1 (top), D2 (middle) and D3 (bottom) are designated by full, striped and dotted lines, respectively. ASVs, amplicon sequence variants; Reads, total number of observed reads

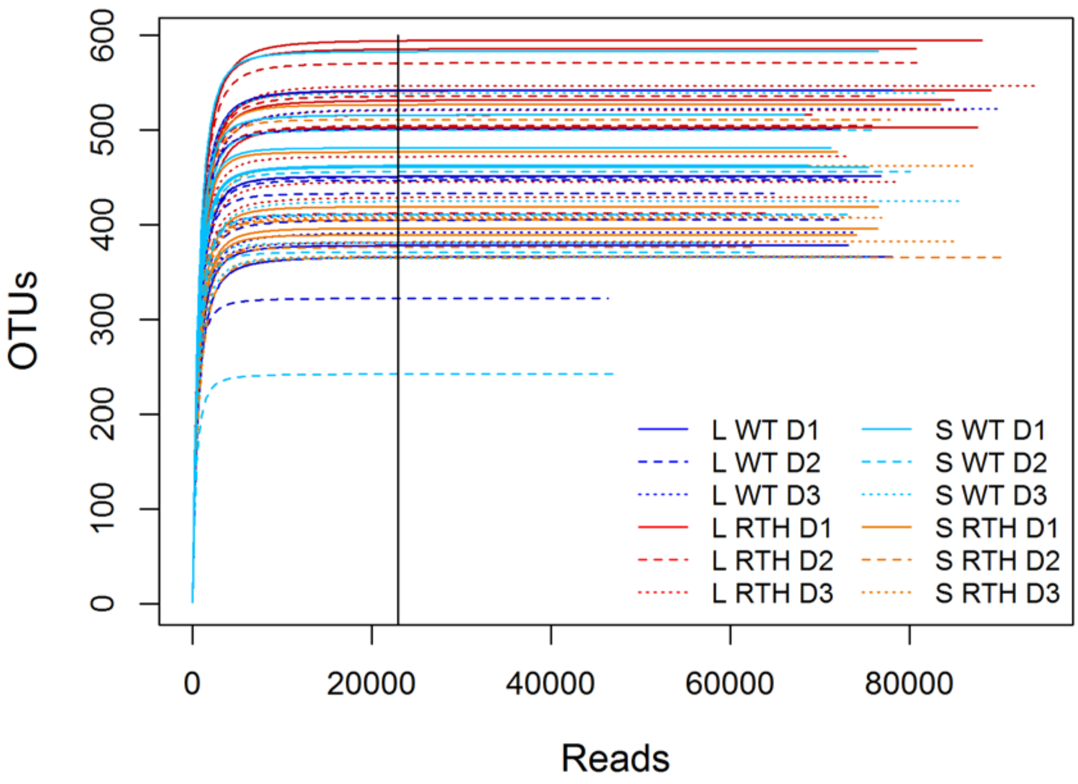

Supplementary Figure S3. Distribution of the *acdS* sequences is most strongly affected by substrate. Principal components analysis plot of *acdS* sequence distribution for maize rhizospheres. Loam, L; sand, S; wild type, WT; *rth3* mutant, RTH; uppermost sampling depth, 1; middle depth, 2; lowest depth, 3

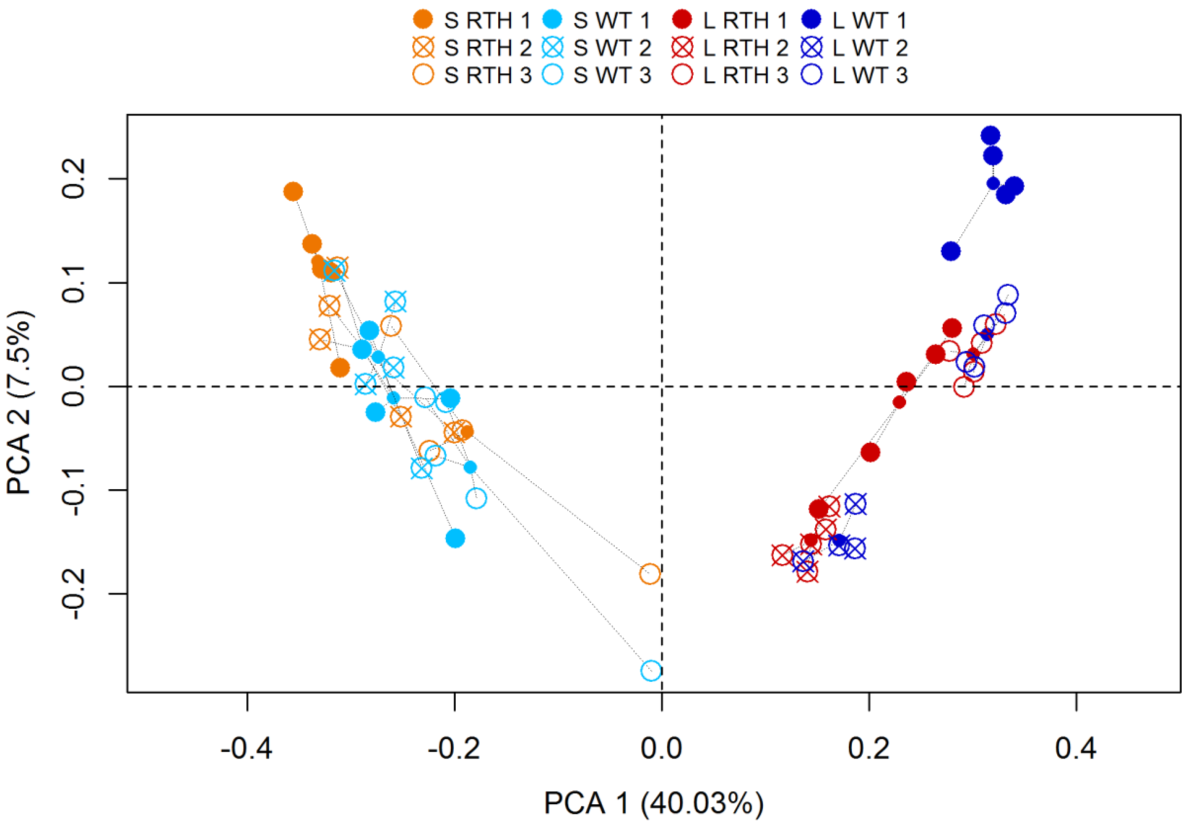

Supplementary Figure S4. Tree of amplicon sequence variants and reference sequences. Tips are collapsed at 98% sequence identity and labelled with the most abundant ASV or a random reference (if there were no ASVs in the set of collapsed tips). Filled inner circles: bootstrap values > 0.75, empty inner circles: bootstrap values > 0.5; grey tip dots: collapsed tips represent only references; green tip dots: collapsed references represent ASVs; grey bar-charts are scaled to the number of represented references per collapsed tip; green bar-charts are scaled to the number of represented ASVs per collapsed tip; tip labels are coloured according to the represented class (blue colors: Actinobacteria; orange-brown colours: Alphaproteobacteria; green colours: Gammaproteobacteria; green-brown: Betaproteobacteria (Burkholderiales)). Higher resolution figure where sequence identities are visible is provided as a separate file Supplementary Figure S4.png

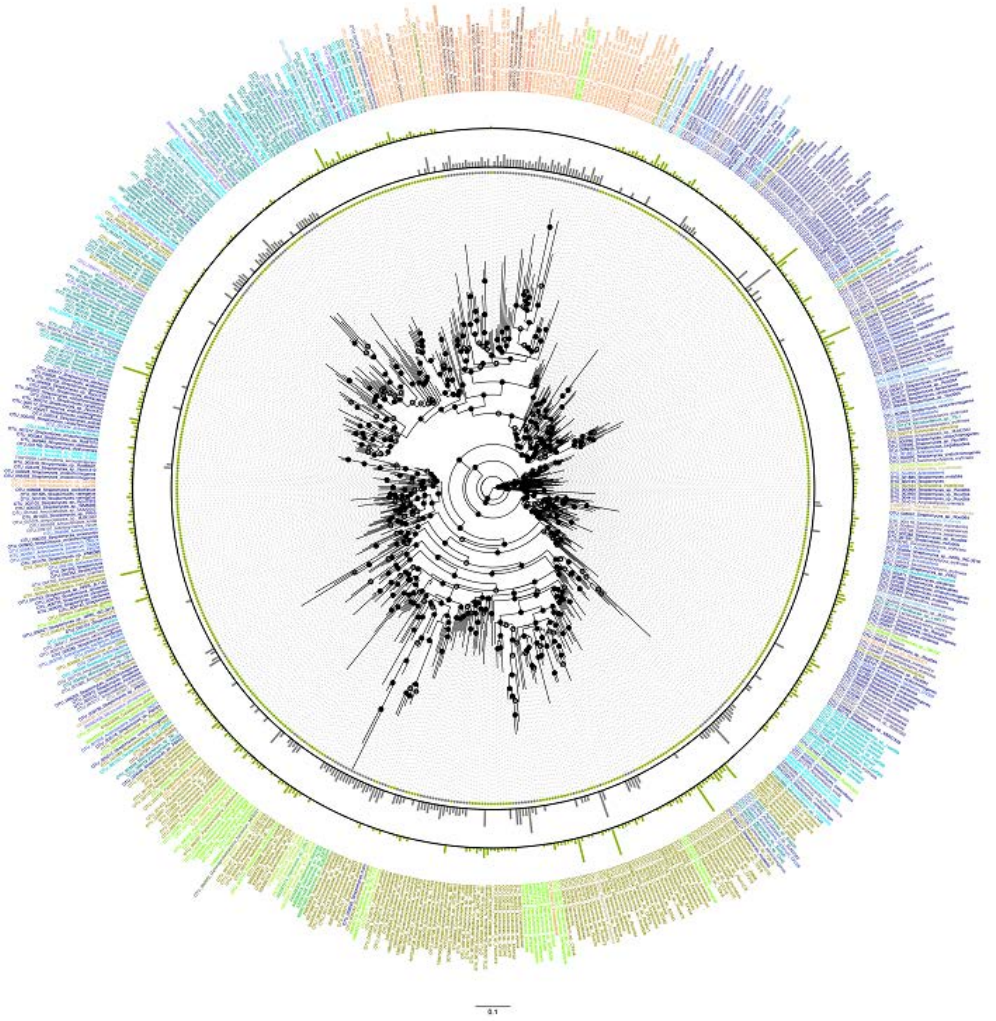

Supplement: Supplementary Figure 1 — Experimental design, maize growth, and water consumption. [file Data_Sheet_1.PDF]
