# Supplementary figures and images for: Soil Texture, Sampling Depth and Root Hairs Shape the Structure of ACC Deaminase Bacterial Community Composition in Maize Rhizosphere
Source: Front Microbiol. 2021 Feb 4;12:616828. doi: 10.3389/fmicb.2021.616828 (PMC7891401; doi:10.3389/fmicb.2021.616828)

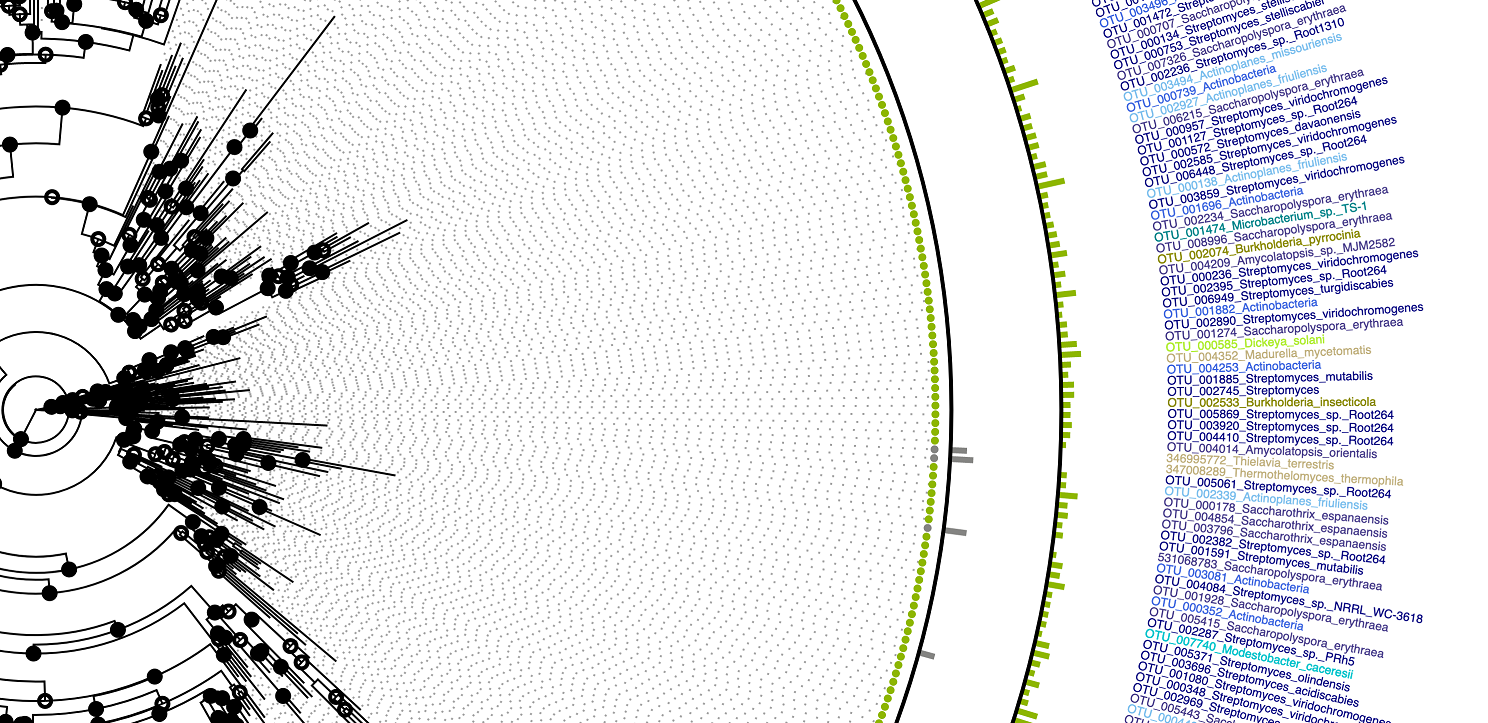

Supplement: Supplementary Figure 4 — Tree of amplicon sequence variants and reference sequences. [file Image_4.png]
